# Challenges and approaches to studying pore-forming proteins

**DOI:** 10.1042/BST20210706

**Published:** 2021-11-08

**Authors:** Joshua T. Benton, Charles Bayly-Jones

**Affiliations:** 1 Department of Biochemistry and Molecular Biology, Monash University, Clayton, Victoria, Australia; 2 Biomedicine Discovery Institute, Faculty of Medicine, Nursing and Health Sciences, Monash University, Clayton, Victoria, Australia

**Keywords:** agricultural bioscience, imaging techniques, membrane proteins, nanotechnology, pore-forming toxins, protein engineering

## Abstract

Pore-forming proteins (PFPs) are a broad class of molecules that comprise various families, structural folds, and assembly pathways. In nature, PFPs are most often deployed by their host organisms to defend against other organisms. In humans, this is apparent in the immune system, where several immune effectors possess pore-forming activity. Furthermore, applications of PFPs are found in next-generation low-cost DNA sequencing, agricultural crop protection, pest control, and biosensing. The advent of cryoEM has propelled the field forward. Nevertheless, significant challenges and knowledge-gaps remain. Overcoming these challenges is particularly important for the development of custom, purpose-engineered PFPs with novel or desired properties. Emerging single-molecule techniques and methods are helping to address these unanswered questions. Here we review the current challenges, problems, and approaches to studying PFPs.

## Introduction

**Figure 1.**
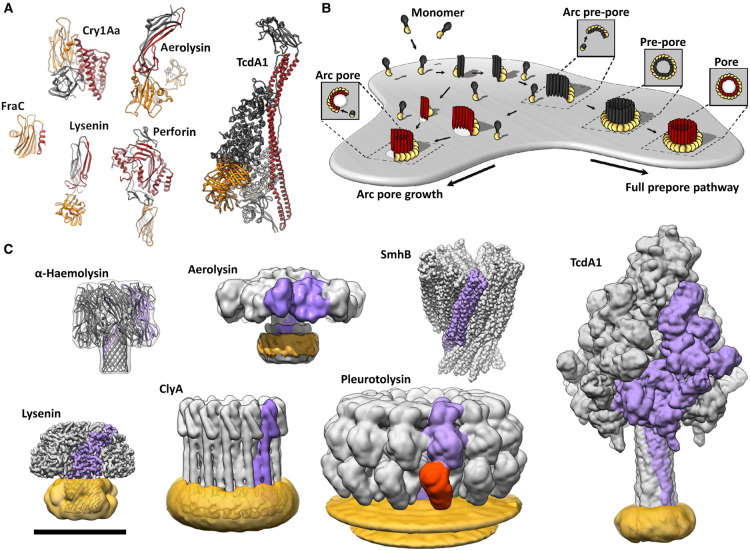
Pore-forming proteins are structurally diverse. (**A**) Protomers of lysenin (RCSB Protein Data Bank [PDB] ID code 3ZXG), *Bacillus thuringiensis* Cry1Aa (PDB: 1CIY), aerolysin (PDB: 3G4N), Fragaceatoxin C (FraC; PDB: 3LIM), perforin (PDB: 3NSJ) and *Photorhabdus luminescens* Tc holotoxin (TcdA1; PDB: 6RW6). Motifs/domains responsible for membrane insertion and membrane binding are coloured red and orange, respectively. (**B**) Schematic depicting a generic pathway for pore formation into a lipid membrane based on MACPF/CDC pore-forming systems. Soluble components, typically monomers, bind the membrane. Several monomers subsequently come together to form oligomers. Oligomeric intermediates undergo a conformational change inserting transmembrane amphipathic regions into the lipid bilayer, forming an aqueous channel. Some other PFPs systems oligomerise in solution before binding the bilayer. Membrane penetration may precede oligomerisation in some systems. The stoichiometry of the various intermediates is highly dependent on the pore-forming system. Additional intermediates (not depicted) may also be present. (**C**) Pore forms (volume rendering in UCSF Chimera) of pleurotolysin (PDB: 4V2T), α-haemolysin (PDB: 3M2L), SmhB (PDB: 7A0G) and ClyA (PDB: 6MRW). Low-pass filtered cryoEM maps of aerolysin (EMDB-8187), lysenin (EMDB-8015) and Photorhabdus luminescens Tc holotoxin (TcdA1; EMDB-10313). A single protomer from each pore is coloured purple (and orange, for the two-component system of pleurotolysin). Where applicable, detergent or lipids are coloured yellow. Scale bar is 100 Å for **A** and **C**.

Pore-forming proteins (PFPs) represent a highly diverse and growing class of molecules identified in all kingdoms of life (Figure 1). Examples include the MACPF/CDC, aerolysin, ClyA, and colicin families, to name a few. PFPs are particularly remarkable in that they transition from a soluble molecule into an integral transmembrane protein. In nature, PFPs function to target and perforate lipid bilayers, often resulting in cell death.

In a typical scenario, a PFP can discriminately bind to a target, whereupon enormous conformational changes take place to insert amphipathic regions into the target bilayer (Figure 1a). These structural changes are often accompanied or preceded by oligomerisation. After assembling into homo- or hetero-oligomers, the inserted amphipathic regions define an aqueous channel or pore. The specific mechanism of pore insertion is highly divergent and not shared by all PFPs. In general, PFPs bind to the membrane, oligomerise, and then insert into the bilayer. However, these steps can occur in varying orders across the different PFP families (Figure 1b).

The function of a PFP is frequently accomplished via a simple arrangement of a receptor-binding domain (RBD) and a pore-forming domain (PFD), although multi-component systems also exist [1–4] (Figure 1a). The RBD can be vastly different and may target specific lipid compositions, protein targets, or even glycans. The PFD is equally diverse and fulfils the role of membrane insertion and often oligomerisation. Once activated, the PFD is capable of completely transitioning from one topological fold to a different one (Figure 1c). The final pore lumen can be as small as a few nanometres (aerolysin) to as large as 50 nm (some CDCs), or larger if multiple arcs conglomerate together.

Broadly, PFPs accomplish biological roles in cell signalling [5–7], programmed cell death [8–10], killing of other cells and organisms [11,12], defence, development [13,14], delivery of effector molecules [15–18], as well as providing a means of digestion. The most obvious function of PFPs — cell killing — is achieved by disrupting the membrane bilayer, resulting in either osmotic shock or diffusion of other effector molecules between membrane compartments. Conversely, cellular signalling and developmental roles are less well understood, representing active areas of research. In lower organisms, PFPs enable bacteria to fight for resources by killing competing species, while in humans, pore-forming immune effectors combat bacterial infections or trigger cell–cell signalling events by damaging cellular membranes [6,19]. To date, numerous families of PFPs have been identified and reviewed extensively (Figure 1a,c) [12,20–28].

The conversion from soluble monomer to integral pores of variable diameter and chemical properties makes PFPs useful in several biotechnology applications. One objective in engineering PFPs is to make tuneable and controllable gateways for the selective flow of compounds, DNA/RNA, and proteins through membranes. In this regard, PFPs are showing promise in the laboratory for DNA sequencing [29,30], proteomics [31], and even compartmentalised biochemical systems [32]. PFPs are also of major interest in industry for their pesticide and antimicrobial properties [33–36], while also being proposed as components in targeted drug-delivery strategies [37,38].

Herein, we review the emerging research themes and challenges in the field. We discuss some of the state-of-the-art techniques being employed to study PFPs and offer a perspective on their contributions in the future. Lastly, we explore some recent examples of biotechnological developments that employ PFPs for translational applications.

## Current questions and challenges

Over the past decade, our understanding of PFP structure and function has been propelled forward by x-ray crystallography and cryoEM studies. In comparison, investigations into pathway kinetics, lipid interactions, and conformational changes of pore formation are relatively rare — representing a major shortcoming in the literature. Here, we outline some of the current challenges and limitations in our understanding of PFPs.

### Identification of conditions for activation

Many novel PFPs have been identified by bioinformatic or high-throughput screening techniques. However, these methods provide little to no information on the conditions required to activate pore formation. Consequently, these conditions remain elusive for many well-studied PFPs. Identifying these conditions is non-trivial and represents a major bottleneck in understanding PFP function — often requiring tedious and labour-intensive screening on a case-by-case basis. Screening can be further complicated if pore formation is triggered by a specific sequence of events. For example, various combinations of triggers have been identified, such as proteolysis [9,10,33,34], changing pH [39–41], receptor binding [17,42–44], encountering lipids of particular compositions [39,41,45–48], and physical properties of the membrane [49].

In the absence of knowledge about native activation conditions, artificial methods have enabled the study of PFPs in their pore form. Successful methods include freeze-thaw treatment [17], incubation with detergents [50], and addition of mild denaturants [51].

### Assembly pathways and kinetics

The assembly pathways that culminate in pore formation are highly diverse and underpin fundamental regulatory and functional attributes specific to a given PFP [52]. Attempts to characterise events along the assembly pathway are met with fundamental challenges. Firstly, intermediates are often short-lived and rapidly transition into different states. Secondly, pore formation is asynchronous, making it difficult to extract mechanistic details from the population averages provided by ensemble analyses.

 In addition to the specific events of assembly, the rates and dynamics of these processes similarly underpin functional and regulatory properties of PFPs. As such, the kinetics of pore formation is a fundamental and complex topic [53–57]. Quantification of these properties yields insight into kinetic bottlenecks for therapeutics, emergent properties of the system, structural and evolutionary constraints of function, and statistical understanding of assembly [52–57].

### Intermediates of pore formation

Excellent progress has been made in determining representative structures of PFPs in various conformational states. These include several stable conformations in the upstream and downstream stages of the pore formation assembly pathway. Notable exceptions include some insecticidal families, such as Cry1 toxins, for which we lack structures of oligomeric species entirely. In comparison, intermediates of pore formation have proven challenging to structurally characterise. These include membrane-bound monomers, small oligomeric assemblies (arcs), early and late prepores, and structures adopted during membrane insertion.

Consequently, studies of intermediate stages of pore formation are rare or require biochemical modifications to the PFP in order to ‘trap’ and visualise intermediates [18,58–64]. Moreover, these modifications also raise questions about the validity of the manipulated state, specifically how closely it reflects a true state in nature, if at all. Furthermore, due to the extensive nature of the rearrangements accompanying pore formation, the simple ‘start-and-end’ structural snapshots provided by these methods leave much to be inferred. As such, detailed atomistic descriptions outlining the conformational changes that occur during pore formation are lacking for many families.

### Lipid properties and biophysics

In addition to being a platform for PFPs to bind and insert into, lipid membranes of different physical properties can also modulate and regulate the function of PFP systems. Intuitively, different lipid headgroups can function to specifically bind and anchor a PFP, as observed for several systems [3,39,41,65,66]. A less intuitive effect on pore formation is that of the physical properties of the bilayer, such as width, density, and fluidity [67]. Even lipid rafts have been implicated in PFP function [68,69]. These properties manifest from the lipid packing, phase transition temperature, saturation of the aliphatic regions, and curvature of the bilayer.

It has recently been demonstrated that the activity of lymphocyte perforin is affected by these properties, with high lipid order and bilayer rigidity negatively regulating function [49]. Furthermore, studies of β-barrel folding kinetics in lipid bilayers have shown a relationship between certain physical properties (width, curvature, composition, fluidity) and the efficiency of β-barrel formation [70–73]. Lipid physical properties likely also modulate the kinetics of PFPs, given differences in protein diffusion rates, membrane binding, sequestration [47], and movement of lipids [74,75] during the formation of a pore.

### Emergent (system-level) properties

Emergent properties, also known as collective behaviour, are system-level characteristics that arise from complex interactions between many constituent parts. These properties are manifest at the system level, but do not belong to the individual components of the system — ‘the whole is greater than the sum'. These include ultrastructure, phase transitions, self-assembly and other macroscopic properties. One example, in the context of PFPs, is the process of self-assembly from monomers into an intricate array of hexagonally arranged prepores, such as the honeycomb arrangement of MPEG1 prepores [39,41].

Understanding and predicting these phenomena is important for designing PFPs systems with desired emergent properties. For example, one might use the honeycomb arrangement of MPEG1 to generate an antimicrobial nanosurface for surgical equipment. Such properties are difficult to predict, however, and even more challenging to design. To achieve these goals, biophysicists require computational and theoretical models based on knowledge of system kinetics, structure and mechanisms that recapitulate and describe PFP behaviour.

## State-of-the-art tools for studying PFPs

**Figure 2.**
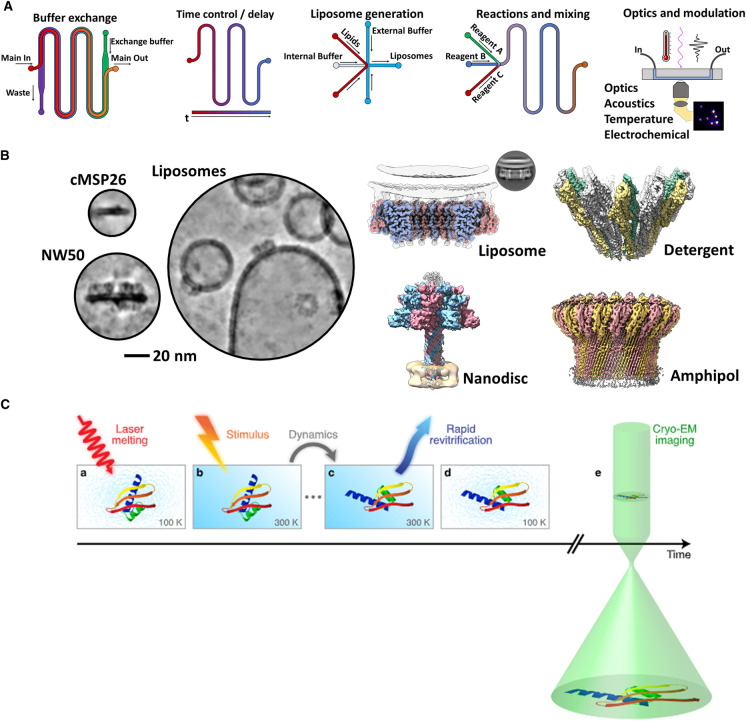
Static and dynamic processes elucidated by cryoEM. (**A**) Microfluidic devices enable complex biochemistry and processes to be performed and studied on-chip. These chips also enable rapid, time-resolved cryoEM. Shown from left to right: microfluidic buffer exchange, time delay for temporal control, liposome generation, mixing of reactions and last, the coupling of detectors/optics to a microfluidic chip for data collection. (**B**) Membrane mimetic technologies enable structural studies of PFPs in a near-native lipid membrane context. Shown are various examples of nanodiscs [15,161–163], liposomes [39,163], detergent [4] and amphipols [88,164] which have all been used to study the pore and prepore forms of PFPs. Select cryoEM examples; MPEG1 [39], XaxAB [4], Anthrax toxin [15] and polyC9 [88] (**C**) Microfluidic and *in situ* time-resolved cryoEM techniques provide structural insight over small increments of time. Panel **C** reproduced from Voss et al. [90]. Stimulation by laser (red) melts the vitreous ice, enabling rapid (photo-dependent; ‘stimulus') conformational changes to occur before re-vitrification. Followed by standard cryoEM imaging (green).

**Figure 3.**
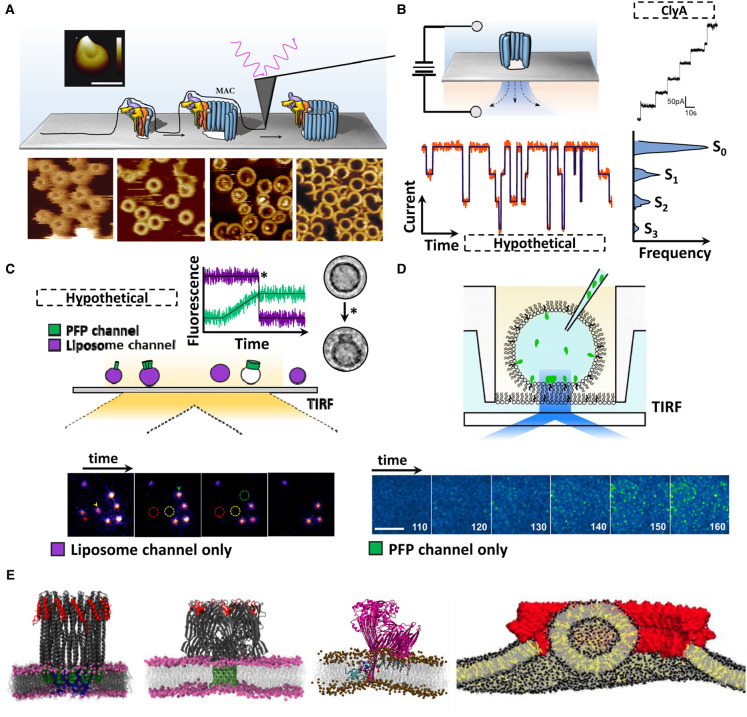
State of the art techniques for studying PFPs. (**A**) Schematic illustration of AFM height topology measurement. Light is reflected off an atomically small tip which traces the assembly of PFPs (MAC; illustrated) in real-time. All AFM images reproduced [39,53,102,104,165]. (**B**) Single-channel conductance measured over a bilayer. Opening-closing of channels (hypothetical trace; orange/purple) reveals different stoichiometric states (histogram; S_0_–S_3_). ClyA example reproduced from Fahie et al. [118]. (**C**) Illustration of single-molecule TIRF microscopy. Immobilised liposomes with an encapsulated fluorescent dye (purple) are tethered to a coverslip. Fluorescently labelled PFPs (green) are flowed over the surface via microfluidics. Accumulation of PFP on the liposome is measured in the PFP channel (not shown), while simultaneously membrane integrity is measured in the liposome channel (four still frames). Pore formation is captured as the sudden loss of fluorescent dye signal upon liposome rupture (circled; Personal correspondence, A/Prof Till Bölking, UNSW). (**D**) Schematic diagram of interface bilayer TIRF microscopy. Below; PFO monomers (green) diffuse on the lipid bilayer over time. Insertion is measured as a sudden loss of diffusion. Modified from Senior et al. [56]. (**E**) Examples of PFP systems constructed and studied *in silico* by MD simulations. Both membrane properties and pore dynamics can be examined (left-most three [atomistic], far right [coarse grain]). Reproduced from Varadarajan et al. Cheerla et al. and Vögele et al. [74,121,122].

Fundamental questions concerning PFP biology can be classified into key research themes, including structural intermediates, kinetics of pore formation, mode-of-action, PFP-lipid interactions, and translational applications in biotechnology and medicine. Driving these investigations are a broad set of cutting-edge experimental and computational techniques (Figures 2, 3). Collectively, these techniques operate within the single-molecule regime. Here, we cover how several techniques have propelled the field forward in recent years.

### Microfluidics

Increasingly, researchers are moving toward high-throughput, parallel, single-molecule based assays that couple microfluidic systems with state-of-the-art techniques (discussed below). The modular nature of microfluidic devices enables complex fluidic circuits to be built in a customisable fashion, integrating liposome fabrication [32,76], protein assays, and small-scale purifications [77] into one (Figure 2a). As such, lab-on-a-chip devices can be developed for specific low-volume, efficient and reproducible experiments for parallel screening, sample optimisation, and data acquisition schemes. To date, microfluidic devices have been developed to perform myriad tasks [76–83]. While only a few studies currently make use of microfluidic or micromanipulation to specifically study PFPs, their use is growing with future applications in high-throughput lipid screening or measuring entire assembly pathways.

### Membrane mimetics & cryoEM

Membrane mimetic technology has drastically accelerated the study of membrane proteins, in conjunction with cryoEM. Membrane mimetics, such as liposomes and nanodiscs, preserve the lipid bilayer while forming in solution (Figure 2b). These offer the select advantage over detergent by acting as a membrane platform upon which PFPs may assemble [16,17,39,58,59,61,84–86]. Membrane mimetics are particularly valuable for interrogating PFP structure in a lipid environment, and as such have been used extensively (Table 1).

**Table 1:** Comparison of various membrane mimetic and substitute technologies

TOOL	ADVANTAGES/BENEFITS	DISADVANTAGES/CHALLENGES	EXAMPLES
LIPOSOMES	Closely mimics native membrane (fluidity, width, phase transitions) Mediates complex assembly Diffusion on surface is possible Encapsulate dyes, reagents, etc. Compartment forming (chemical gradients, assays, etc) Easily deposited onto a surface Simple to produce, size is controllable.	Instability - prone to burst or aggregate Heterogeneous (composition, protein binding, size) Not effective for solubilisation Difficult to freeze, requires thick ice and complicates high-resolution cryoEM reconstruction (strong incoherent signal) Large enough to scatter light Screening lipid compositions is low throughput	CryoEM (1–4) AFM (5,6) SCC (7) LM (8)
NANODISCS MSP1D1 cMSP26 NW50	Highly stable Can derive lipids native bilayers Smaller than liposomes (reducing ice thickness for cryoEM studies) Stabilisation of hydrophobic regions (like detergent) Mediates complex assembly Amenable to further purification Genetically encoded allowing customisation Click-chemistry compatible	Lipids in nanodiscs have altered properties compared to lipids in cell membranes Low surface area (diffusion dependent process) Production and purification are labour intensive Often requires extensive optimisation Poor efficiency of nanodisc formation, not effective for solubilisation Sizes are relatively limited	CryoEM (9–13)
DETERGENT	Simple and convenient means of solubilisation A broad selection with different chemistries Can induce spontaneous oligomerisation and/or pore formation Amenable to further purification	Do not provide a surface for assembly Detergent micelles are topologically different to native membranes Can introduce structural artefacts Protein stability is often an issue Lack protein/lipid interactions Empty micelles can interfere with cryoEM image processing	CryoEM (14–20)
AMPHIPOLS	Less likely to destabilise structure compared to detergent Stable, even when diluted Can be chemically modified or conjugated No interference with light-based experiments	Does not provide an assembly surface Not suitable at acidic pH or in excessive divalent ions. Cannot directly solubilise MPs Difficult to synthesis Polydisperse	CryoEM (21–24)
SMALPS	Detergent-free solubilisation of MPs. Maintain native lipids after solubilisation. Useful for lipidomics. Protein-lipids interactions are retained to a greater extent Compatible with further purification Highly stable once SMA disc forms Does not form micelles	Difficult to synthesise Polydisperse sizes Native bilayer properties are not preserved. Expensive Can affect protein purification	CryoEM (25) MS (26) SCC (27)
PEPTIDISC & SAPOSIN	Diameter of the disc is dependent on the protein diameter Works at low pH Genetically encoded Easy to produce in large quantities	No assembly surface Does not preserve bilayer properties	
*IN SITU*	Direct observation of sample in native environment	Low resolution Low throughput Technically complex	CryoET (28,29) AFM (30,31) SCC (32)
SUPPORTED LIPID BILAYERS	Enables visualisation of diffusion dependent processes Large surface area Simple and readily applicable to AFM, SPR, TIRF, etc.	Lipid diffusion is significantly reduced compared to native bilayers Can easily study and visualise phase transition properties	AFM (5,6,33–35) LM (36–38)

Detergents have also been widely used to study pores by cryoEM and x-ray crystallography [50,84,87]. Some PFP systems spontaneously form prepore and pore oligomers in the presence of detergents [50]. While for other PFPs, detergents have been used to extract pores from native membranes or stabilise activated pores *in vitro* [41,84,85,87].

Amphipathic polymers, such as amphipols or styrene maleic acid polymers (SMALPs), offer an alternative to detergents to extract and/or stabilise membrane proteins. While SMALPs have not been used yet to study PFPs, amphipols have been used to stabilise large oligomeric pores [88]. For the readers’ convenience, we have summarised the advantages and disadvantages of various membrane mimetics and their applications (Table 1).

### Time-resolved cryoEM

In general, structural studies provide only a static snapshot (Figure 2b). Recently, new methods in cryoEM demonstrate the potential for time-resolved measurements and ‘4D reconstructions'. Progress toward this goal has been made in sample preparation, microscope hardware, data collection, and analysis techniques [79,80,89–97].

The most established example is the development of rapid microfluidic freezing devices, which have enabled millisecond mixing and halting of biochemical reactions before standard cryoEM imaging [93]. Conversely, a new cutting-edge approach achieves microsecond temporal increments by devitrifying the specimen for a controlled period in the microscope using a laser pulse (Figure 2c) [90]. During this devitrified period the specimen can undergo rapid conformational changes before undergoing re-vitrification. This treatment is followed by standard cryoEM imaging.

Lastly, modern software can now estimate the conformational landscape from an ensemble of single particles in some cases [89,94–97]. In this context, trajectories through the conformational landscape reflect temporal changes of the macromolecule in real space. In this regard, these trajectories can be considered a form of pseudo-time-resolved analysis. Taken together these techniques provide insight into a new dimension of structure and function. We anticipate these methods to be valuable in probing short-lived (∼µs–ms) structural intermediates of PFPs in coming years, especially when combined with machine learning techniques [98,99].

### Atomic force microscopy (AFM)

Time-resolved cryoEM is still in its infancy. In comparison, AFM is a mature technique capable of directly measuring the assembly pathway of a PFP at moderate-to-high spatial and temporal resolutions [67,100] (Figure 3a). In the past, AFM has been used to provide insight into both the oligomeric state(s) of PFPs and their dynamics over time, as well as specific details about PFP activation in the context of lipid membranes [67,100,101]. Indeed, AFM has been used to establish the foundation of mechanistic and structural understanding of various systems, including MACPF/CDCs [53,59,102,103], GSDMs [104], and others [105,106].

Recently, AFM studies have provided both single-molecule kinetics of assembly [53], as well as an understanding of bactericidal activity by imaging pore formation on live cells [107,108]. AFM has been especially useful for studying the interaction of PFPs with lipid bilayers, and particularly the functional impact membrane properties have on PFPs [49]. Modern AFM instruments are becoming increasingly capable of achieving both high temporal and spatial resolutions, especially when combined with new approaches and algorithms [109,110]. With improvements in modern instrumentation and new algorithms, we envisage an AFM ‘resolution revolution' to be on the horizon.

### Single-molecule fluorescence microscopy

Unlike AFM, a major advantage of fluorescence microscopy is the ability to follow multiple differently labelled components at comparable temporal resolutions (or higher). As such, fluorescence microscopy, particularly single-molecule modalities, has been used extensively for PFPs [57]. Indeed, smFRET and TIRF studies have been employed to study various aspects of PFP dynamics and kinetics. These include measurements of complex assembly, stoichiometry, diffusion coefficients, single-molecule kinetics and lipid membrane interactions [47,52,54,56,111,112]. Evidently, single-molecule TIRF microscopy is a versatile tool to study multiple stages of pore formation and probe the mechanism of assembly at high temporal resolution (Figure 3c,d).

### Single-channel conductance

**Figure 4.**
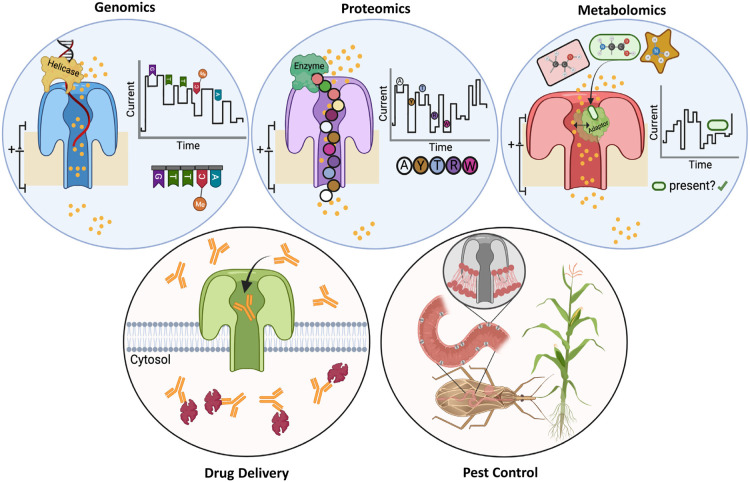
Translational applications of pore forming protein. Top row, left to right. Pore-forming proteins (PFPs) are used in next-generation DNA sequencing. DNA is passed through a nanopore (blue) in the presence of an electric current generated by the flow of ions (yellow circles). Characteristic changes in current can be mapped to particular nucleobases (including methylated cytosines [Me]). Similarly, nanopore technology has been applied to proteomics (purple), allowing discrimination of peptides with single amino acid changes. Proteomic nanopores are often coupled with an enzyme (green), such as an unfoldase or helicase, that threads the peptide through the pore. Nanopores are also used in metabolomics to detect small molecules. A nanopore (red) can capture an adaptor protein (green) that binds to a specific metabolite e.g., green amino acid. Metabolite binding induces a conformational change in the adaptor protein, leading to a characteristic change in ion flow through the nanopore. Bottom row, left to right. PFPs have been used to deliver antibodies into mammalian cancer cells. Nanopores (green) provide a passage for antibodies (orange) to cross the cell membrane and bind to their intracellular target (red). PFPs are also a staple pest control agent in agriculture, helping to protect crops from lepidopteran pests. PFPs from the crop (grey) are ingested by the pest, and subsequently perforate cells lining the pest's digestive system. Created with BioRender.com.

Single-channel conductance (SCC) is another powerful and ubiquitous technique used to study individual nanopores [113–118] (Figure 3b). By establishing an electric potential across an impermeable bilayer, the presence of a membrane disruption (due to an arc or pore) can be detected by current flow. By measuring the steps and transient drops in current, their duration and amplitude, various details of the underlying molecular process can be extracted. This includes the size distribution of the nanopore, interactions with solutes or binding partners (e.g. Vip1/2), as well as pore assembly pathways and kinetics in the context of systems that form pores via a growing-arc mechanism (Figure 1b) [113–117]. Indeed, SCC forms the technological foundation of new polymer sequencing methods (see applications below). Individual nucleotides or amino acids can be detected by small blockages of nanopores, which produce a characteristic fingerprint as a function of their physical properties, like charge and size (Figure 4).

### Molecular dynamics simulations

Historically, molecular dynamics simulations have enabled investigation of length and time scales not accessible to common experimental techniques (Figure 3e). Atomistic simulations of PFPs have revealed flexible hinge regions [119], protein/lipid interactions [47,74,120], and the distortion or reorganisation of lipid bilayers [75,121,122]. Furthermore, coarse-grained simulations have enabled larger oligomeric assemblies to be studied over longer time scales to investigate initial stages of oligomerisation (Figure 3e). Computational models of PFPs extend beyond structure. Indeed complex interaction pathways and kinetic processes can be modelled mathematically and simulated to reveal the temporal evolution of the system [55]. Additionally, computation simulations have been advantageous in translational research in studying electrical fields and current flow of nanopores [114,123]. While not a comprehensive list, these examples do show how simulations provide a powerful tool to understand atomistic details, quaternary interactions, and complex, system-level emergent properties of PFPs.

## Efforts to re-engineer & modulate PFPs

Applications for PFPs have emerged in both academic and industrial settings. With nature providing a range of pore scaffolds to harness, re-engineering efforts have aimed to optimise pore properties for specific purposes. These include next-generation DNA sequencing technologies, which correlate subtle changes in the flow of ions through a nanopore with the translocation of nucleotide sequences (Figure 4). PFPs commonly used for DNA sequencing possess a narrow constriction point(s) (∼1 nm) within their lumen [115,124]. As DNA is threaded through the pore and encounters these narrow regions, the pore becomes partially blocked — reducing current flow by a characteristic amount for each nucleotide combination. Re-engineering constriction sites (and other residues lining the pore lumen) by point mutations has greatly improved sequencing accuracy [125,126]. More radical changes to pore architecture have also been made, including truncation and covalent/non-covalent addition of adaptor proteins or enzymes [127–129]. Re-engineered PFPs fill several useful niches in DNA/RNA sequencing, including long-read sequencing [130–132], in-field metagenomics [133–135], and even epigenetics [136–140].

There has also been work to extend nanopore-based technology to various proteomic applications, including mass identification [117,123,141,142], peptide, and protein sequencing [143–147] (Figure 4). While nanopore-based proteomics promises extremely high sensitivity, and even the possibility of single-cell proteomics [31], sequencing polypeptides remains a frontier challenge in PFP engineering. In the case of *de novo* peptide sequencing, difficulties lie in translocating chemically diverse peptides of a non-uniform charge density through the pore and deciphering each of the 20 amino acids. By trapping individual peptides within the wild-type aerolysin pore, it was possible to distinguish between peptides with single residue substitutions for 13 of the 20 amino acids [147]. However, progress is still required to sequentially ‘read’ individual residues while passing a peptide through a nanopore.

Another challenge in nanopore proteomics is that protein sequencing requires unfolding and regular ratcheting of the denatured polypeptide chain through the pore. Partnering pores with other enzymes, such as unfoldases, helicases, DNA polymerases, and proteasomes has allowed for polypeptide translocation [144,146,148,149]. However, there is currently an insufficient level of control over single-pass translocation for accurate residue assignment. To address this, the use of a DNA-peptide conjugate enabled repeated re-reading of peptides, improving discrimination between a limited set of peptide variants [144]. While several challenges exist, proteomic nanopores could eventually be used on-bench to detect protein point variants, cleavage events, or post-translational modifications — all at the single-molecule level [123].

Besides nanopore sequencing, PFPs have also been co-opted for metabolomics — allowing the detection of specific ligands, such as sugars, amino acids, and vitamins [150–152] (Figure 4). Such systems rely on the pore capturing an adaptor protein capable of recognising a specific ligand, with the binding of the ligand to the adaptor generating a distinct change in the nanopore's ion conductivity. In the future, nanopores may be routinely used for real-time metabolomics to monitor human health [150], or even in analytical devices for industrial applications [153].

As drug and cargo delivery systems, PFPs are being developed to transport various effector molecules into cells [154,155] (Figure 4). For example, clostridium protective antigen toxin (CPAT) has recently been modified to inject antibodies and other therapeutic molecules into mammalian cells in a controlled and specific manner, thereby circumventing the membrane barrier [37,38,156]. As such, PFPs may represent a novel class of drug delivery system.

Meanwhile in agriculture, PFPs derived from the entomopathogenic bacteria, *Bacillus thuringiensis*, are used in transgenic crops as a key defence against pests, such as lepidopteran species (Figure 4)*.* These PFPs therefore help to safeguard various essential commodities such as corn, soy, and cotton by successfully controlling crop pests — saving billions of dollars annually [35,157,158]. While hugely successful, new PFPs are required to target emerging pests and to combat the increasingly problematic rise in resistance to existing transgenic crops.

The applications of PFPs mentioned throughout this review have relied on pre-existing pores that have been adapted to suit particular contexts. Eventually, it may be practical to *de novo* design PFPs for specific purposes — allowing complete control over pore properties. In fact, *de novo* design of simple α and β transmembrane channels was recently achieved [159,160], potentially laying the foundation for tailor-made PFPs designed for specific analytes and applications.

## Concluding remarks

The field of PFPs is a remarkably diverse and rich area of investigation, covering kinetics, structures to fieldwork and proteomics. Recent developments in technology underpin several advances in the field of PFPs. Increasingly, researchers are relying on advanced techniques and integrative studies to provide insight into the biological role and function of PFPs. Specifically, microfluidic devices and single-molecule techniques are gaining greater traction and providing deeper functional insight into various systems. Researchers are finding novel and exciting areas in which translational applications of PFPs can benefit academia, industry, and biomedicine. It is our perspective that the rational *de novo* design or re-engineering of custom PFPs is the next frontier in the field of PFPs.

## Perspectives

- PFPs are a broad class of molecules which underpin biological processes in immunology, pathogenicity, development and cell signalling. Furthermore, translational applications of PFPs can be found in biomedicine, agriculture and biotechnologies such as DNA and protein sequencing.
- Single-molecule based imaging and spectroscopic technologies are transforming the understanding of molecular processes and mechanisms that govern PFP behaviour and function.
- Improved structural, kinetic and mechanistic understanding of PFPs coupled with cutting-edge computational methods provide a tangible foundation for the next frontier of research in PFPs — *de novo* design of custom PFP systems for biotechnology and biomedicine.

## Open Access Statement

Open access for this article was enabled by the participation of Monash University in an all-inclusive Read & Publish pilot with Portland Press and the Biochemical Society under a transformative agreement with CAUL.

## Abbreviations

AFM: Atomic force microscopy
CDC: cholesterol dependent cytolysin
LM: light microscopy
MAC: Membrane Attack Complex
MACPF: Membrane Attack Complex/Perforin
MD: Molecular dynamics
MS: mass spectrometry
PFD: Pore forming domain
PFO: Perfringolysin O
PFP: Pore forming protein
RBD: Receptor binding domain
SCC: Single channel conductance
SMALPs: Styrene maleic acid copolymer lipid particles
smFRET: Single molecule Forster Resonance Energy Transfer
SPR: surface plasmon resonance
TIRF: Total internal reflection fluorescence
(TR-)CryoEM: (Time-resolved) cryogenic electron microscopy

## References

[1] Giglio, M.L., Ituarte, S., Milesi, V., Dreon, M.S., Brola, T.R., Caramelo, J. et al. (2020) Exaptation of two ancient immune proteins into a new dimeric pore-forming toxin in snails. J. Struct. Biol. 211, 107531 10.1016/j.jsb.2020.107531 32446810

[2] Schnepf, H.E., Lee, S., Dojillo, J.A., Burmeister, P., Fencil, K., Morera, L. et al. (2005) Characterization of Cry34/Cry35 binary insecticidal proteins from diverse *Bacillus thuringiensis* strain collections. Appl. Environ. Microbiol. 71, 1765–1774 10.1128/AEM.71.4.1765-1774.2005 15811999 PMC1082557

[3] Tomita, T., Noguchi, K., Mimuro, H., Ukaji, F., Ito, K., Sugawara-Tomita, N. et al. (2004) Pleurotolysin, a novel sphingomyelin-specific two-component cytolysin from the edible mushroom *Pleurotus ostreatus*, assembles into a transmembrane pore complex. J. Biol. Chem. 279, 26975–26982 10.1074/jbc.M402676200 15084605

[4] Schubert, E., Vetter, I.R., Prumbaum, D., Penczek, P.A. and Raunser, S. (2018) Membrane insertion of α-xenorhabdolysin in near-atomic detail. eLife [Internet] 7, e38017 10.7554/eLife.38017 30010541 PMC6086661

[5] Hung, L.-Y., Tanaka, Y., Herbine, K., Pastore, C., Singh, B., Ferguson, A. et al. (2020) Cellular context of IL-33 expression dictates impact on anti-helminth immunity. Sci. Immunol. 5, eabc6259 10.1126/sciimmunol.abc6259 33188058 PMC8257082

[6] McCormack, R.M., Hunte, R., Podack, E.R., Plano G, V. and Shembade, N. (2020) An essential role for perforin-2 in type I IFN signaling. J. Immunol. 204, 2242–2256 10.4049/jimmunol.1901013 32161097

[7] Halperin, J.A., Taratuska, A. and Nicholson-Weller, A. (1993) Terminal complement complex C5b-9 stimulates mitogenesis in 3T3 cells. J. Clin. Invest. 91, 1974–1978 10.1172/JCI116418 8486768 PMC288194

[8] Sborgi, L., Rühl, S., Mulvihill, E., Pipercevic, J., Heilig, R., Stahlberg, H. et al. (2016) GSDMD membrane pore formation constitutes the mechanism of pyroptotic cell death. EMBO J. 35, 1766–1778 10.15252/embj.201694696 27418190 PMC5010048

[9] Rogers, C., Fernandes-Alnemri, T., Mayes, L., Alnemri, D., Cingolani, G. and Alnemri, E.S. (2017) Cleavage of DFNA5 by caspase-3 during apoptosis mediates progression to secondary necrotic/pyroptotic cell death. Nat. Commun. 8, 14128 10.1038/ncomms14128 28045099 PMC5216131

[10] Shi, J., Zhao, Y., Wang, K., Shi, X., Wang, Y., Huang, H. et al. (2015) Cleavage of GSDMD by inflammatory caspases determines pyroptotic cell death. Nature 526, 660–665 10.1038/nature15514 26375003

[11] Hamon, M.A., Ribet, D., Stavru, F. and Cossart, P. (2012) Listeriolysin O: the Swiss army knife of listeria. Trends Microbiol. 20, 360–368 10.1016/j.tim.2012.04.006 22652164

[12] Popoff, M.R. (2014) Clostridial pore-forming toxins: Powerful virulence factors. Anaerobe 30, 220–238 10.1016/j.anaerobe.2014.05.014 24952276

[13] Berkowicz, S.R., Giousoh, A. and Bird, P.I. (2017) Neurodevelopmental MACPFs: The vertebrate astrotactins and BRINPs. Semin. Cell Dev. Biol. 72, 171–181 10.1016/j.semcdb.2017.05.005 28506896

[14] Johnson, T.K., Crossman, T., Foote, K.A., Henstridge, M.A., Saligari, M.J., Forbes Beadle, L. et al. (2013) Torso-like functions independently of torso to regulate drosophila growth and developmental timing. Proc. Natl Acad. Sci. U.S.A. 110, 14688–14692 10.1073/pnas.1309780110 23959885 PMC3767542

[15] Machen, A.J., Fisher, M.T. and Freudenthal, B.D. (2021) Anthrax toxin translocation complex reveals insight into the lethal factor unfolding and refolding mechanism. Sci. Rep. 11, 13038 [Internet] Available from: http://www.nature.com/articles/s41598-021-91596-3 34158520 10.1038/s41598-021-91596-3 PMC8219829

[16] Roderer, D., Hofnagel, O., Benz, R. and Raunser, S. (2019) Structure of a Tc holotoxin pore provides insights into the translocation mechanism. Proc. Natl Acad. Sci. U.S.A. 116, 23083–23090 10.1073/pnas.1909821116 31666324 PMC6859359

[17] Piper, S.J., Brillault, L., Rothnagel, R., Croll, T.I., Box, J.K., Chassagnon, I. et al. (2019) Cryo-EM structures of the pore-forming A subunit from the yersinia entomophaga ABC toxin. Nat. Commun. 10, 1952 [Internet] http://www.nature.com/articles/s41467-019-09890-8 31028251 10.1038/s41467-019-09890-8 PMC6486591

[18] Anderson, D.M., Sheedlo, M.J., Jensen, J.L. and Lacy, D.B. (2020) Structural insights into the transition of Clostridioides difficile binary toxin from prepore to pore. Nat. Microbiol. 5, 102–107 10.1038/s41564-019-0601-8 31712627 PMC6925320

[19] Morgan, B.P. (2016) The membrane attack complex as an inflammatory trigger. Immunobiology 221, 747–751 10.1016/j.imbio.2015.04.006 25956457

[20] Li, Y., Li, Y., Mengist, H.M., Shi, C., Zhang, C., Wang, B. et al. (2021) Structural basis of the pore-forming toxin/membrane interaction. Toxins (Basel) 13, 128 10.3390/toxins13020128 33572271 PMC7914777

[21] Lacomel, C.J., Dunstone, M.A. and Spicer, B.A. (2021) Branching out the aerolysin, ETX/MTX-2 and toxin_10 family of pore forming proteins. J. Invertebr. Pathol., 107570 10.1016/j.jip.2021.107570 33775676

[22] Anderluh, G., Kisovec, M., Kraševec, N. and Gilbert, R.J.C. (2014) MACPF/CDC proteins—agents of defence, attack and invasion. Subcell. Biochem. 80, 7–30 10.1007/978-94-017-8881-6_2 24798005

[23] Rosado, C.J., Kondos, S., Bull, T.E., Kuiper, M.J., Law, R.H.P., Buckle, A.M. et al. (2008) The MACPF/CDC family of pore-forming toxins. Cell Microbiol. 10, 1765–1774 10.1111/j.1462-5822.2008.01191.x 18564372 PMC2654483

[24] Roderer, D. and Raunser, S. (2019) Tc toxin complexes: assembly, membrane permeation, and protein translocation. Annu. Rev. Microbiol. 73, 247–265 10.1146/annurev-micro-102215-095531 31140906

[25] Peraro, M.D. and van der Goot, F.G. (2015) Pore-forming toxins: ancient, but never really out of fashion. Nat. Rev. Microbiol. 14, 77–92 10.1038/nrmicro.2015.3 26639780

[26] Liu, X. and Lieberman, J. (2020) Knocking ‘em dead: pore-forming proteins in immune defense. Annu. Rev. Immunol. 38, 455–485 10.1146/annurev-immunol-111319-023800 32004099 PMC7260445

[27] Krawczyk, P.A., Laub, M. and Kozik, P. (2020) To kill but not be killed: controlling the activity of mammalian pore-forming proteins. Front. Immunol. 11, 601405 10.3389/fimmu.2020.601405 33281828 PMC7691655

[28] Palma, L., Muñoz, D., Berry, C., Murillo, J., Caballero, P. and Caballero, P. (2014) Bacillus thuringiensis toxins: An overview of their biocidal activity. Toxins 6, 3296–3325 10.3390/toxins6123296 25514092 PMC4280536

[29] Haque, F., Li, J., Wu, H.C., Liang, X.J. and Guo, P. (2013) Solid-state and biological nanopore for real-time sensing of single chemical and sequencing of DNA. Nano Today 8, 56–74 10.1016/j.nantod.2012.12.008 23504223 PMC3596169

[30] Branton, D., Deamer, D.W., Marziali, A., Bayley, H., Benner, S.A., Butler, T. et al. (2008) The potential and challenges of nanopore sequencing. Nat. Biotechnol. 26, 1146–1153 10.1038/nbt.1495 18846088 PMC2683588

[31] Alfaro, J.A., Bohländer, P., Dai, M., Filius, M., Howard, C.J., van Kooten, X.F. et al. (2021) The emerging landscape of single-molecule protein sequencing technologies. Nat. Methods 18, 604–617 10.1038/s41592-021-01143-1 34099939 PMC8223677

[32] Deshpande, S., Brandenburg, F., Lau, A., Last, M.G.F., Spoelstra, W.K., Reese, L. et al. (2019) Spatiotemporal control of coacervate formation within liposomes. Nat. Commun. 10, 1800 10.1038/s41467-019-09855-x 30996302 PMC6470218

[33] Byrne, M.J., Iadanza, M.G., Perez, M.A., Maskell, D.P., George, R.M., Hesketh, E.L. et al. (2021) Cryo-EM structures of an insecticidal Bt toxin reveal its mechanism of action on the membrane. Nat. Commun. 12, 2791 10.1038/s41467-021-23146-4 33990582 PMC8121907

[34] Núñez-Ramírez, R., Huesa, J., Bel, Y., Ferré, J., Casino, P. and Arias-Palomo, E. (2020) Molecular architecture and activation of the insecticidal protein Vip3Aa from bacillus thuringiensis. Nat. Commun. 11, 3974 10.1038/s41467-020-17758-5 32769995 PMC7414852

[35] Hutchison, W.D., Burkness, E.C., Mitchell, P.D., Moon, R.D., Leslie, T.W., Fleischer, S.J. et al. (2010) Areawide suppression of European corn borer with Bt maize reaps savings to non-Bt maize growers. Science 330, 222–225 10.1126/science.1190242 20929774

[36] Badran, A.H., Guzov, V.M., Huai, Q., Kemp, M.M., Vishwanath, P., Kain, W. et al. (2016) Continuous evolution of *Bacillus thuringiensis* toxins overcomes insect resistance. Nature 533, 58–63 10.1038/nature17938 27120167 PMC4865400

[37] Liao, X., Rabideau, A.E. and Pentelute, B.L. (2014) Delivery of antibody mimics into mammalian cells via anthrax toxin protective antigen. ChemBioChem 15, 2458–2466 10.1002/cbic.201402290 25250705 PMC4498471

[38] Rabideau, A.E., Liao, X., Akçay, G. and Pentelute, B.L. (2015) Translocation of non-canonical polypeptides into cells using protective antigen. Sci. Rep. 5, 11944 10.1038/srep11944 26178180 PMC4503955

[39] Pang, S.S., Bayly-Jones, C., Radjainia, M., Spicer, B.A., Law, R.H.P., Hodel, A.W. et al. (2019) The cryo-EM structure of the acid activatable pore-forming immune effector macrophage-expressed gene 1. Nat. Commun. 10, 1–9 [Internet] 10.1038/s41467-019-12279-2 PMC6753088 31537793

[40] Schuerch, D.W., Wilson-Kubalek, E.M. and Tweten, R.K. (2005) Molecular basis of listeriolysin O pH dependence. Proc. Natl Acad. Sci. U.S.A. 102, 12537–12542 10.1073/pnas.0500558102 16105950 PMC1194900

[41] Ni, T., Jiao, F., Yu, X., Aden, S., Ginger, L., Williams, S.I. et al. (2020) Structure and mechanism of bactericidal mammalian perforin-2, an ancient agent of innate immunity. Sci. Adv. 6, 1–13 10.1126/sciadv.aax8286 PMC6989145 32064340

[42] Giddings, K.S.K., Zhao, J., Sims, P.P.J. and Tweten, R.K.R. (2004) Human CD59 is a receptor for the cholesterol-dependent cytolysin intermedilysin. Nat. Struct. Mol. Biol. 11, 1173–1178 10.1038/nsmb862 15543155

[43] Roderer, D., Bröcker, F., Sitsel, O., Kaplonek, P., Leidreiter, F., Seeberger, P.H. et al. (2020) Glycan-dependent cell adhesion mechanism of Tc toxins. Nat. Commun. 11, 2694 10.1038/s41467-020-16536-7 32483155 PMC7264150

[44] Sato, R., Adegawa, S., Li, X., Tanaka, S. and Endo, H. (2019) Function and role of ATP-binding cassette transporters as receptors for 3D-cry toxins. Toxins (Basel) 11, 124 10.3390/toxins11020124 PMC6409751 30791434

[45] Ota, K., Leonardi, A., Mikelj, M., Skočaj, M., Wohlschlager, T., Künzler, M. et al. (2013) Membrane cholesterol and sphingomyelin, and ostreolysin A are obligatory for pore-formation by a MACPF/CDC-like pore-forming protein, pleurotolysin B. Biochimie 95, 1855–1864 10.1016/j.biochi.2013.06.012 23806422

[46] Endapally, S., Frias, D., Grzemska, M., Gay, A., Tomchick, D.R. and Radhakrishnan, A. (2019) Molecular discrimination between Two conformations of sphingomyelin in plasma membranes. Cell 176, 1040–1053.e17 10.1016/j.cell.2018.12.042 30712872 PMC6428426

[47] Sathyanarayana, P., Maurya, S., Behera, A., Ravichandran, M., Visweswariah, S.S., Ayappa, K.G. et al. (2018) Cholesterol promotes Cytolysin A activity by stabilizing the intermediates during pore formation. Proc. Natl Acad. Sci. U.S.A. 115, E7323–E7330 10.1073/pnas.1721228115 30012608 PMC6077711

[48] Ruan, J., Xia, S., Liu, X., Lieberman, J. and Wu, H. (2018) Cryo-EM structure of the gasdermin A3 membrane pore. Nature 557, 62–67 10.1038/s41586-018-0058-6 29695864 PMC6007975

[49] Rudd-Schmidt, J.A., Hodel, A.W., Noori, T., Lopez, J.A., Cho, H.J., Verschoor, S. et al. (2019) Lipid order and charge protect killer T cells from accidental death. Nat. Commun. 10, 5396 10.1038/s41467-019-13385-x 31776337 PMC6881447

[50] Peng, W., de Souza Santos, M., Li, Y., Tomchick, D.R. and Orth, K. (2019) High-resolution cryo-EM structures of the *E. coli* hemolysin ClyA oligomers. PLoS ONE 14, e0213423 [Internet] https://dx.plos.org/10.1371/journal.pone.0213423 31048915 10.1371/journal.pone.0213423 PMC6497250

[51] Katayama, H., Janowiak, B.E., Brzozowski, M., Juryck, J., Falke, S., Gogol, E.P. et al. (2008) GroEL as a molecular scaffold for structural analysis of the anthrax toxin pore. Nat. Struct. Mol. Biol. 15, 754–760 10.1038/nsmb.1442 18568038 PMC2504863

[52] Subburaj, Y., Ros, U., Hermann, E., Tong, R. and García-Sáez, A.J. (2015) Toxicity of an α-pore-forming toxin depends on the assembly mechanism on the target membrane as revealed by single molecule imaging. J. Biol. Chem. 290, 4856–4865 10.1074/jbc.M114.600676 25525270 PMC4335225

[53] Parsons, E.S., Stanley, G.J., Pyne, A.L.B., Hodel, A.W., Nievergelt, A.P., Menny, A. et al. (2019) Single-molecule kinetics of pore assembly by the membrane attack complex. Nat. Commun. 10, 2066 10.1038/s41467-019-10058-7 31061395 PMC6502846

[54] Thompson, J.R., Cronin, B., Bayley, H. and Wallace, M.I. (2011) Rapid assembly of a multimeric membrane protein pore. Biophys. J. 101, 2679–2683 10.1016/j.bpj.2011.09.054 22261056 PMC3297801

[55] Lee, A.A., Senior, M.J., Wallace, M.I., Woolley, T.E. and Griffiths, I.M. (2016) Dissecting the self-assembly kinetics of multimeric pore-forming toxins. J. R. Soc. Interface 13, 20150762 10.1098/rsif.2015.0762 26763328 PMC4759788

[56] Senior, M.J., Monico, C., Weatherill, E.E., Gilbert, R.J., Heuck, A.P. and Wallace, M.I. (2021) Single-molecule imaging of cholesterol-dependent cytolysin assembly. bioRxiv [Internet] http://biorxiv.org/content/early/2021/05/27/2021.05.26.445776.abstract

[57] Parperis, C. and Wallace, M.I. (2021) Single-molecule imaging of pore-forming toxin dynamics in droplet interface bilayers. In: Methods in Enzymology 649, 431–459 [Internet] https://linkinghub.elsevier.com/retrieve/pii/S0076687921000574 10.1016/bs.mie.2021.01.035 33712195

[58] Lukoyanova, N., Kondos, S.C., Farabella, I., Law, R.H.P., Reboul, C.F., Caradoc-Davies, T.T. et al. (2015) Conformational changes during pore formation by the perforin-related protein pleurotolysin. PLoS Biol. 13, 1–15 10.1371/journal.pbio.1002049 PMC4318580 25654333

[59] Leung, C., Dudkina, N.V., Lukoyanova, N., Hodel, A.W., Farabella, I., Pandurangan, A.P. et al. (2014) Stepwise visualization of membrane pore formation by suilysin, a bacterial cholesterol-dependent cytolysin. eLife 3, e04247 [Internet] http://elifesciences.org/content/early/2014/12/02/eLife.04247.abstract 25457051 10.7554/eLife.04247 PMC4381977

[60] Hotze, E.M., Wilson-kubalek, E.M., Rossjohn, J., Parker, M.W., Johnson, A.E. and Tweten, R.K. (2001) Arresting pore formation of a cholesterol-dependent cytolysin by disulfide trapping synchronizes the insertion of the transmembrane β-sheet from a prepore intermediate. J. Biol. Chem. 276, 8261–8268 10.1074/jbc.M009865200 11102453

[61] Shah, N.R., Voisin, T.B., Parsons, E.S., Boyd, C.M., Hoogenboom, B.W. and Bubeck, D. (2020) Structural basis for tuning activity and membrane specificity of bacterial cytolysins. Nat. Commun. 11, 5818 10.1038/s41467-020-19482-6 33199689 PMC7669874

[62] Wade, K.R., Hotze, E.M., Kuiper, M.J., Morton, C.J., Parker, M.W., Tweten, R.K. et al. (2015) An intermolecular electrostatic interaction controls the prepore-to-pore transition in a cholesterol-dependent cytolysin. Proc. Natl Acad. Sci. U.S.A. 112, 2204–2209 10.1073/pnas.1423754112 25646411 PMC4343107

[63] Tsitrin, Y., Morton, C.J., El Bez, C., Paumard, P., Velluz, M.C., Adrian, M. et al. (2002) Conversion of a transmembrane to a watersoluble protein complex by a single point mutation. Nat. Struct. Biol. 9, 729–733 10.1038/nsb839 12219082

[64] Iacovache, I., De Carlo, S., Cirauqui, N., Dal Peraro, M., van der Goot, F.G. and Zuber, B. (2016) Cryo-EM structure of aerolysin variants reveals a novel protein fold and the pore-formation process. Nat. Commun. 7, 12062 10.1038/ncomms12062 27405240 PMC4947156

[65] Hodel, A.W., Rudd-Schmidt, J., Trapani, J.A., Voskoboinik, I. and Hoogenboom, B. (2021) Lipid specificity of the immune effector perforin. Faraday Discuss 10.1039/d0fd00043d PMC8704153 34545865

[66] Athanasiadis, A., Anderluh, G., Maček, P. and Turk, D. (2001) Crystal structure of the soluble form of equinatoxin II, a pore-forming toxin from the sea anemone actinia equina. Structure 9, 341–346 10.1016/S0969-2126(01)00592-5 11525171

[67] Hodel, A.W., Hammond, K. and Hoogenboom, B.W. (2021) AFM imaging of pore forming proteins. Methods Enzymol. 649, 149–188 10.1016/bs.mie.2021.01.002 33712186

[68] Raghunathan, K., Foegeding, N.J., Campbell, A.M., Cover, T.L., Ohi, M.D. and Kenworthy, A.K. (2018) Determinants of raft partitioning of the helicobacter pylori pore-forming toxin vacA. Infect. Immun. 86, e00872-17 10.1128/IAI.00872-17 29531133 PMC5913846

[69] Guo, X.-L., Liu, L.-Z., Wang, Q.-Q., Liang, J.-Y., Lee, W.-H., Xiang, Y. et al. (2019) Endogenous pore-forming protein complex targets acidic glycosphingolipids in lipid rafts to initiate endolysosome regulation. Commun. Biol. 2, 59 10.1038/s42003-019-0304-y 30775460 PMC6370762

[70] Danoff, E.J. and Fleming, K.G. (2015) Membrane defects accelerate outer membrane β-barrel protein folding. Biochemistry 54, 97–99 10.1021/bi501443p 25513891 PMC4303321

[71] Danoff, E.J. and Fleming, K.G. (2017) Novel kinetic intermediates populated along the folding pathway of the transmembrane β-barrel OmpA. Biochemistry 56, 47–60 10.1021/acs.biochem.6b00809 28001375 PMC5826654

[72] Burgess, N.K., Dao, T.P., Stanley, A.M. and Fleming, K.G. (2008) β-Barrel proteins that reside in the *Escherichia coli* outer membrane in vivo demonstrate varied folding behavior in vitro. J. Biol. Chem. 283, 26748–26758 10.1074/jbc.M802754200 18641391 PMC3258919

[73] Gessmann, D., Chung, Y.H., Danoff, E.J., Plummer, A.M., Sandlin, C.W., Zaccai, N.R. et al. (2014) Outer membrane β-barrel protein folding is physically controlled by periplasmic lipid head groups and BamA. Proc. Natl Acad. Sci. U.S.A. 111, 5878–5883 10.1073/pnas.1322473111 24715731 PMC4000854

[74] Vögele, M., Bhaskara, R.M., Mulvihill, E., van Pee, K., Yildiz, Ö., Kühlbrandt, W. et al. (2019) Membrane perforation by the pore-forming toxin pneumolysin. Proc. Natl Acad. Sci. U.S.A. 116, 13352–7 10.1073/pnas.1904304116 31209022 PMC6613103

[75] Ponmalar, I.I., Cheerla, R., Ayappa, K.G. and Basu, J.K. (2019) Correlated protein conformational states and membrane dynamics during attack by pore-forming toxins. Proc. Natl Acad. Sci. U.S.A. 116, 12839–12844 [Internet] http://www.pnas.org/lookup/doi/10.1073/pnas.1821897116 31189600 10.1073/pnas.1821897116 PMC6600976

[76] Deshpande, S. and Dekker, C. (2018) On-chip microfluidic production of cell-sized liposomes. Nat. Protoc. 13, 856–874 10.1038/nprot.2017.160 29599442

[77] Schmidli, C., Albiez, S., Rima, L., Righetto, R., Mohammed, I., Oliva, P. et al. (2019) Microfluidic protein isolation and sample preparation for high-resolution cryo-EM. Proc. Natl Acad. Sci. U.S.A. 116, 15007–15012 10.1073/pnas.1907214116 31292253 PMC6660723

[78] Nagamine, K., Onodera, S., Torisawa, Y.S., Yasukawa, T., Shiku, H. and Matsue, T. (2005) On-chip transformation of bacteria. Anal. Chem. 77, 4278–4281 10.1021/ac048278n 15987137

[79] Mäeots, M.-E., Lee, B., Nans, A., Jeong, S.-G., Esfahani, M.M.N., Ding, S. et al. (2020) Modular microfluidics enables kinetic insight from time-resolved cryo-EM. Nat. Commun. 11, 3465 10.1038/s41467-020-17230-4 32651368 PMC7351747

[80] Feng, X., Fu, Z., Kaledhonkar, S., Jia, Y., Shah, B., Jin, A. et al. (2017) A fast and effective microfluidic spraying-plunging method for high-resolution single-particle cryo-EM. Structure 25, 663–670.e3 10.1016/j.str.2017.02.005 28286002 PMC5382802

[81] Convery, N. and Gadegaard, N. (2019) 30 years of microfluidics. Micro Nano Eng. 2, 76–91 10.1016/j.mne.2019.01.003

[82] Sanchez-Freire, V., Ebert, A.D., Kalisky, T., Quake, S.R. and Wu, J.C. (2012) Microfluidic single-cell real-time PCR for comparative analysis of gene expression patterns. Nat. Protoc. 7, 829–838 10.1038/nprot.2012.021 22481529 PMC3657501

[83] Arter, W.E., Levin, A., Krainer, G. and Knowles, T.P.J. (2020) Microfluidic approaches for the analysis of protein–protein interactions in solution. Biophys. Rev. 12, 575–585 10.1007/s12551-020-00679-4 32266673 PMC7242286

[84] Menny, A., Serna, M., Boyd, C.M., Gardner, S., Joseph, A.P., Morgan, B.P. et al. (2018) CryoEM reveals how the complement membrane attack complex ruptures lipid bilayers. Nat. Commun. 9, 5316 10.1038/s41467-018-07653-5 30552328 PMC6294249

[85] Serna, M., Giles, J.L., Morgan, B.P. and Bubeck, D. (2016) Structural basis of complement membrane attack complex formation. Nat. Commun. 7, 10587 10.1038/ncomms10587 26841837 PMC4743022

[86] van Pee, K., Neuhaus, A., D'Imprima, E., Mills, D.J., Kühlbrandt, W. and Yildiz, Ö. (2017) CryoEM structures of membrane pore and prepore complex reveal cytolytic mechanism of Pneumolysin. eLife 6, e23644 10.7554/eLife.23644 28323617 PMC5437283

[87] Bokori-Brown, M., Martin, T.G., Naylor, C.E., Basak, A.K., Titball, R.W. and Savva, C.G. (2016) Cryo-EM structure of lysenin pore elucidates membrane insertion by an aerolysin family protein. Nat. Commun. 7, 11293 [Internet] http://www.nature.com/articles/ncomms11293 27048994 10.1038/ncomms11293 PMC4823867

[88] Spicer, B.A., Law, R.H.P., Caradoc-davies, T.T., Ekkel, S.M., Bayly-Jones, C., Pang, S. et al. (2018) The first transmembrane region of complement component-9 acts as a brake on its self-assembly. Nat. Commun. 9, 3266 [Internet] 10.1038/s41467-018-05717-0 30111885 PMC6093860

[89] Zhong, E.D., Bepler, T., Berger, B. and Davis, J.H. (2021) CryoDRGN: reconstruction of heterogeneous cryo-EM structures using neural networks. Nat. Methods 18, 176–185 10.1038/s41592-020-01049-4 33542510 PMC8183613

[90] Voss, J.M., Harder, O.F., Olshin, P.K., Drabbels, M. and Lorenz, U.J. (2021) Rapid melting and revitrification as an approach to microsecond time-resolved cryo-electron microscopy. Chem. Phys. Lett. 778, 138812 10.1016/j.cplett.2021.138812

[91] Frank, J. (2017) Time-resolved cryo-electron microscopy: Recent progress. J. Struct. Biol. 200, 303–306 10.1016/j.jsb.2017.06.005 28625887 PMC5732889

[92] Dandey, V.P., Budell, W.C., Wei, H., Bobe, D., Maruthi, K., Kopylov, M. et al. (2020) Time-resolved cryo-EM using spotiton. Nat. Methods 17, 897–900 10.1038/s41592-020-0925-6 32778833 PMC7799389

[93] Fu, Z., Kaledhonkar, S., Borg, A., Sun, M., Chen, B., Grassucci, R.A. et al. (2016) Key intermediates in ribosome recycling visualized by time-resolved cryoelectron microscopy. Structure 24, 2092–2101 10.1016/j.str.2016.09.014 27818103 PMC5143168

[94] Chen, M. and Ludtke, S.J. (2021) Deep learning-based mixed-dimensional Gaussian mixture model for characterizing variability in cryo-EM. Nat. Methods 18, 930–936 10.1038/s41592-021-01220-5 34326541 PMC8363932

[95] Dashti, A., Mashayekhi, G., Shekhar, M., Ben Hail, D., Salah, S., Schwander, P. et al. (2020) Retrieving functional pathways of biomolecules from single-particle snapshots. Nat. Commun. 11, 4734 10.1038/s41467-020-18403-x 32948759 PMC7501871

[96] Punjani, A. and Fleet, D.J. (2021) 3D variability analysis: Resolving continuous flexibility and discrete heterogeneity from single particle cryo-EM. J. Struct. Biol. 213, 107702 10.1016/j.jsb.2021.107702 33582281

[97] Punjani, A. and Fleet, D.J. (2021) 3D flexible refinement: structure and motion of flexible proteins from Cryo-EM. bioRxiv 10.1101/2021.04.22.440893 PMC10250194 37169929

[98] Baek, M., DiMaio, F., Anishchenko, I., Dauparas, J., Ovchinnikov, S., Lee, G.R. et al. (2021) Accurate prediction of protein structures and interactions using a three-track neural network. Science 373, eabj8754 10.1126/science.abj8754 PMC7612213 34282049

[99] Jumper, J., Evans, R., Pritzel, A., Green, T., Figurnov, M., Ronneberger, O. et al. (2021) Highly accurate protein structure prediction with alphaFold. Nature 596, 583–589 10.1038/s41586-021-03819-2 34265844 PMC8371605

[100] Jiao, F., Ruan, Y. and Scheuring, S. (2021) High-speed atomic force microscopy to study pore-forming proteins. In: Methods in Enzymology 649, 189–217 10.1016/bs.mie.2021.01.033 33712187

[101] Unsay, J.D. and García-Sáez, A.J. (2019) AFM to study pore-forming proteins. Methods Mol. Biol. 1886, 191–202 10.1007/978-1-4939-8894-5_10 30374868

[102] Leung, C., Hodel, A.W., Brennan, A.J., Lukoyanova, N., Tran, S., House, C.M. et al. (2017) Real-time visualization of perforin nanopore assembly. Nat. Nanotechnol. 12, 467–473 10.1038/nnano.2016.303 28166206

[103] Ruan, Y., Rezelj, S., Bedina Zavec, A., Anderluh, G. and Scheuring, S. (2016) Listeriolysin O membrane damaging activity involves arc formation and lineaction: implication for listeria monocytogenes escape from phagocytic vacuole. PLoS Pathog. 12, e1005597 10.1371/journal.ppat.1005597 27104344 PMC4841516

[104] Mulvihill, E., Sborgi, L., Mari, S.A., Pfreundschuh, M., Hiller, S. and Müller, D.J. (2018) Mechanism of membrane pore formation by human gasdermin-D. EMBO J. 37, e98321 10.15252/embj.201798321 29898893 PMC6043855

[105] Munguira, I.L.B., Takahashi, H., Casuso, I. and Scheuring, S. (2017) Lysenin toxin membrane insertion is pH-dependent but independent of neighboring lysenins. Biophys. J. 113, 2029–2036 10.1016/j.bpj.2017.08.056 29117526 PMC5685674

[106] Morante, K., Bellomio, A., Gil-Cartón, D., Redondo-Morata, L., Sot, J., Scheuring, S. et al. (2016) Identification of a membrane-bound prepore species clarifies the lytic mechanism of actinoporins. J. Biol. Chem. 291, 19210–9 10.1074/jbc.M116.734053 27445331 PMC5016661

[107] Doorduijn, D.J., Bardoel, B.W., Heesterbeek, D.A.C., Ruyken, M., Benn, G., Parsons, E.S. et al. (2020) Bacterial killing by complement requires direct anchoring of membrane attack complex precursor C5b-7. PLoS Pathog. 16, e1008606 10.1371/journal.ppat.1008606 32569291 PMC7351214

[108] Benn, G., Pyne, A.L.B., Ryadnov, M.G. and Hoogenboom, B.W. (2019) Imaging live bacteria at the nanoscale: comparison of immobilisation strategies. Analyst 144, 6944–6952 10.1039/C9AN01185D 31620716 PMC7138128

[109] Heath, G.R., Kots, E., Robertson, J.L., Lansky, S., Khelashvili, G., Weinstein, H. et al. (2021) Localization atomic force microscopy. Nature 594, 385–390 10.1038/s41586-021-03551-x 34135520 PMC8697813

[110] Heath, G.R. and Scheuring, S. (2018) High-speed AFM height spectroscopy reveals µs-dynamics of unlabeled biomolecules. Nat. Commun. 9, 4983 10.1038/s41467-018-07512-3 30478320 PMC6255864

[111] Benke, S., Roderer, D., Wunderlich, B., Nettels, D., Glockshuber, R. and Schuler, B. (2015) The assembly dynamics of the cytolytic pore toxin clyA. Nat. Commun. 6, 6198 10.1038/ncomms7198 25652783 PMC4347018

[112] Rojko, N., Cronin, B., Danial, J.S.H., Baker, M.A.B., Anderluh, G. and Wallace, M.I. (2014) Imaging the lipid-phase-dependent pore formation of equinatoxin II in droplet interface bilayers. Biophys J. 106, 1630–1637 10.1016/j.bpj.2013.11.4507 24739162 PMC4008837

[113] Rojko, N., Kristan, K.Č, Viero, G., Žerovnik, E., Maček, P., Dalla Serra, M. et al. (2013) Membrane damage by an α-helical pore-forming protein, equinatoxin II, proceeds through a succession of ordered steps. J. Biol. Chem. 288, 23704–23715 10.1074/jbc.M113.481572 23803608 PMC3745318

[114] Huang, G., Willems, K., Bartelds, M., van Dorpe, P., Soskine, M. and Maglia, G. (2020) Electro-Osmotic vortices promote the capture of folded proteins by PlyAB nanopores. Nano Lett. 20, 3819–3827 10.1021/acs.nanolett.0c00877 32271587 PMC7227020

[115] Crnković, A., Srnko, M. and Anderluh, G. (2021) Biological nanopores: Engineering on demand. Life 11, 27 10.3390/life11010027 33466427 PMC7824896

[116] Diederichs, T., Pugh, G., Dorey, A., Xing, Y., Burns, J.R., Hung Nguyen, Q. et al. (2019) Synthetic protein-conductive membrane nanopores built with DNA. Nat. Commun. 10, 5018 10.1038/s41467-019-12639-y 31685824 PMC6828756

[117] Huang, G., Voet, A. and Maglia, G. (2019) Frac nanopores with adjustable diameter identify the mass of opposite-charge peptides with 44 dalton resolution. Nat. Commun. 10, 835 10.1038/s41467-019-08761-6 30783102 PMC6381162

[118] Fahie, M., Romano, F.B., Chisholm, C., Heuck, A.P., Zbinden, M. and Chen, M. (2013) A Non-classical assembly pathway of *Escherichia coli* pore-forming toxin cytolysin A. J. Biol. Chem. 288, 31042–31051 10.1074/jbc.M113.475350 24019520 PMC3829417

[119] Reboul, C.F., Whisstock, J.C. and Dunstone, M.A. (2014) A New model for pore formation by cholesterol-dependent cytolysins. PLoS Comput. Biol. 10, e1003791 10.1371/journal.pcbi.1003791 25144725 PMC4140638

[120] Desikan, R., Behera, A., Maiti, P.K. and Ayappa, K.G. (2021) Using multiscale molecular dynamics simulations to obtain insights into pore forming toxin mechanisms. Methods Enzymol. 649, 461–502 10.1016/bs.mie.2021.01.021 33712196

[121] Varadarajan, V., Desikan, R. and Ayappa, K.G. (2020) Assessing the extent of the structural and dynamic modulation of membrane lipids due to pore forming toxins: insights from molecular dynamics simulations. Soft Matter. 16, 4840–4857 10.1039/D0SM00086H 32421131

[122] Cheerla, R. and Ayappa, K.G. (2020) Molecular dynamics study of lipid and cholesterol reorganization due to membrane binding and pore formation by listeriolysin O. J. Membr. Biol. 253, 535–550 10.1007/s00232-020-00148-9 33118046

[123] Huang, G., Willems, K., Soskine, M., Wloka, C. and Maglia, G. (2017) Electro-osmotic capture and ionic discrimination of peptide and protein biomarkers with fraC nanopores. Nat. Commun. 8, 935 10.1038/s41467-017-01006-4 29038539 PMC5715100

[124] Zhou, W., Qiu, H., Guo, Y. and Guo, W. (2020) Molecular insights into distinct detection properties of α-hemolysin, mspA, csgG, and aerolysin nanopore sensors. J. Phys. Chem. B 124, 1611–1618 10.1021/acs.jpcb.9b10702 32027510

[125] Stoddart, D., Heron, A.J., Klingelhoefer, J., Mikhailova, E., Maglia, G. and Bayley, H. (2010) Nucleobase recognition in ssDNA at the central constriction of the α-hemolysin pore. Nano Lett. 10, 3633–3637 10.1021/nl101955a 20704324 PMC2935931

[126] Butler, T.Z., Pavlenok, M., Derrington, I.M., Niederweis, M. and Gundlach, J.H. (2008) Single-molecule DNA detection with an engineered MspA protein nanopore. Proc. Natl Acad. Sci. U.S.A. 105, 20647–20652 10.1073/pnas.0807514106 19098105 PMC2634888

[127] Ayub, M., Stoddart, D. and Bayley, H. (2015) Nucleobase recognition by truncated α-Hemolysin pores. ACS Nano 9, 7895–7903 10.1021/nn5060317 26114210 PMC4830132

[128] Van der Verren, S.E., Van Gerven, N., Jonckheere, W., Hambley, R., Singh, P., Kilgour, J. et al. (2020) A dual-constriction biological nanopore resolves homonucleotide sequences with high fidelity. Nat. Biotechnol. 38, 1415–1420 10.1038/s41587-020-0570-8 32632300 PMC7610451

[129] Wang, S., Zhao, Z., Haque, F. and Guo, P. (2018) Engineering of protein nanopores for sequencing, chemical or protein sensing and disease diagnosis. Curr. Opin. Biotechnol. 51, 80–89 10.1016/j.copbio.2017.11.006 29232619 PMC5994363

[130] Jain, M., Koren, S., Miga, K.H., Quick, J., Rand, A.C., Sasani, T.A. et al. (2018) Nanopore sequencing and assembly of a human genome with ultra-long reads. Nat. Biotechnol. 36, 338–345 10.1038/nbt.4060 29431738 PMC5889714

[131] Sakamoto, Y., Sereewattanawoot, S. and Suzuki, A. (2020) A new era of long-read sequencing for cancer genomics. J. Hum. Genet. 65, 3–10 10.1038/s10038-019-0658-5 31474751 PMC6892365

[132] Karst, S.M., Ziels, R.M., Kirkegaard, R.H., Sørensen, E.A., McDonald, D., Zhu, Q. et al. (2021) High-accuracy long-read amplicon sequences using unique molecular identifiers with nanopore or pacBio sequencing. Nat. Methods 18, 165–169 10.1038/s41592-020-01041-y 33432244

[133] Latorre-Pérez, A., Villalba-Bermell, P., Pascual, J. and Vilanova, C. (2020) Assembly methods for nanopore-based metagenomic sequencing: a comparative study. Sci. Rep. 10, 13588 10.1038/s41598-020-70491-3 32788623 PMC7423617

[134] Kafetzopoulou, L.E., Pullan, S.T., Lemey, P., Suchard, M.A., Ehichioya, D.U., Pahlmann, M. et al. (2019) Metagenomic sequencing at the epicenter of the Nigeria 2018 Lassa fever outbreak. Science 363, 74–77 10.1126/science.aau9343 30606844 PMC6855379

[135] Edwards, A., Debbonaire, A., Nicholls, S., Rassner, S., Sattler, B., Cook, J. et al. (2016) In-field metagenome and 16S rRNA gene amplicon nanopore sequencing robustly characterize glacier microbiota. bioRxiv 10.1101/073965

[136] Akbari, V., Garant, J.-M., O'Neill, K., Pandoh, P., Moore, R., Marra, M.A. et al. (2021) Genome-wide detection of imprinted differentially methylated regions using nanopore sequencing. bioRxiv 10.1101/2021.07.17.452734 PMC9255983 35787786

[137] Gershman, A., Sauria, M.E.G. Hook, P.W., Hoyt, S.J., Razaghi, R., Koren, S. et al. (2021) Epigenetic patterns in a complete human genome. bioRxiv 10.1101/2021.05.26.443420 PMC9170183 35357915

[138] Laszlo, A.H., Derrington, I.M., Brinkerhoff, H., Langford, K.W., Nova, I.C., Samson, J.M. et al. (2013) Detection and mapping of 5-methylcytosine and 5-hydroxymethylcytosine with nanopore MspA. Proc. Natl Acad. Sci. U.S.A. 110, 18904–9 10.1073/pnas.1310240110 24167255 PMC3839702

[139] Schreiber, J., Wescoe, Z.L., Abu-Shumays, R., Vivian, J.T., Baatar, B., Karplus, K. et al. (2013) Error rates for nanopore discrimination among cytosine, methylcytosine, and hydroxymethylcytosine along individual DNA strands. Proc. Natl Acad. Sci. U.S.A. 110, 18910–5 10.1073/pnas.1310615110 24167260 PMC3839712

[140] Silva, C., Machado, M., Ferrão, J., Rodrigues, S. and Vieira, L.) Whole human genome 5′-mC methylation analysis using long read nanopore sequencing. bioRxiv 10.1101/2021.05.20.444035 PMC9665147 35856633

[141] Chavis, A.E., Brady, K.T., Hatmaker, G.A., Angevine, C.E., Kothalawala, N., Dass, A. et al. (2017) Single molecule nanopore spectrometry for peptide detection. ACS Sensors 2, 1319–1328 10.1021/acssensors.7b00362 28812356 PMC11274829

[142] Robertson, J.W.F., Rodrigues, C.G., Stanford, V.M., Rubinson, K.A., Krasilnikov O, V. and Kasianowicz, J.J. (2007) Single-molecule mass spectrometry in solution using a solitary nanopore. Proc. Natl Acad. Sci. U.S.A. 104, 8207–8211 10.1073/pnas.0611085104 17494764 PMC1866312

[143] Ayub, M. and Bayley, H. (2016) Engineered transmembrane pores. Curr. Opin. Chem. Biol. 34, 117–126 10.1016/j.cbpa.2016.08.005 27658267 PMC5123773

[144] Brinkerhoff, H., Kang, A.S.W., Liu, J., Aksimentiev, A. and Dekker, C. (2021) Infinite re-reading of single proteins at single-amino-acid resolution using nanopore sequencing. bioRxiv 10.1101/2021.07.13.452225 PMC8811723 34735217

[145] Howorka, S. and Siwy, Z.S. (2020) Reading amino acids in a nanopore. Nat. Biotechnol. 38, 159–160 10.1038/s41587-019-0401-y 31974421

[146] Nivala, J., Mulroney, L., Li, G., Schreiber, J. and Akeson, M. (2014) Discrimination among protein variants using an unfoldase-coupled nanopore. ACS Nano 8, 12365–12375 10.1021/nn5049987 25402970

[147] Ouldali, H., Sarthak, K., Ensslen, T., Piguet, F., Manivet, P., Pelta, J. et al. (2020) Electrical recognition of the twenty proteinogenic amino acids using an aerolysin nanopore. Nat. Biotechnol. 38, 176–181 10.1038/s41587-019-0345-2 31844293 PMC7008938

[148] Zhang, S., Huang, G., Versloot, R., Herwig, B.M., de Souza, P.C.T., Marrink, S.J. et al. (2020) Bottom-up fabrication of a multi-component nanopore sensor that unfolds, processes and recognizes single proteins. bioRxiv 10.1101/2020.12.04.411884

[149] Yan, S., Zhang, J., Wang, Y., Guo, W., Zhang, S., Liu, Y. et al. (2021) Single molecule ratcheting motion of peptides in a *Mycobacterium smegmatis* Porin A (MspA) nanopore. Nano Lett. 21, 6703–6710 10.1021/acs.nanolett.1c02371 34319744

[150] Zernia, S., Van Der Heide, N.J., Galenkamp, N.S., Gouridis, G. and Maglia, G. (2020) Current blockades of proteins inside nanopores for real-time metabolome analysis. ACS Nano 14, 2296–2307 10.1021/acsnano.9b09434 32003969 PMC7045694

[151] Galenkamp, N.S., Soskine, M., Hermans, J., Wloka, C. and Maglia, G. (2018) Direct electrical quantification of glucose and asparagine from bodily fluids using nanopores. Nat. Commun. 9, 4085 10.1038/s41467-018-06534-1 30291230 PMC6173770

[152] Haque, F., Lunn, J., Fang, H., Smithrud, D. and Guo, P. (2012) Real-Time sensing and discrimination of single chemicals using the channel of Phi29 DNA packaging nanomotor. ACS Nano 6, 3251–3261 https://pubs.acs.org/doi/10.1021/nn3001615 22458779 10.1021/nn3001615 PMC3337346

[153] Bétermier, F., Cressiot, B., Di Muccio, G., Jarroux, N., Bacri, L., della Rocca B, M. et al. (2020) Single-sulfur atom discrimination of polysulfides with a protein nanopore for improved batteries. Commun. Mater. 1, 59 10.1038/s43246-020-00056-4

[154] Roderer, D., Schubert, E., Sitsel, O. and Raunser, S. (2019) Towards the application of Tc toxins as a universal protein translocation system. Nat. Commun. 10, 5263 10.1038/s41467-019-13253-8 31748551 PMC6868009

[155] Lu, Z., Truex, N.L., Melo, M.B., Cheng, Y., Li, N., Irvine, D.J. et al. (2021) IgG-Engineered protective antigen for cytosolic delivery of proteins into cancer cells. ACS Cent. Sci. 7, 365–378 10.1021/acscentsci.0c01670 33655074 PMC7908032

[156] Loftis, A.R., Santos, M.S., Truex, N.L., Biancucci, M., Satchell, K.J.F. and Pentelute, B.L. (2020) Anthrax protective antigen retargeted with single-chain variable fragments delivers enzymes to pancreatic cancer cells. ChemBioChem 21, 2772–2776 10.1002/cbic.202000201 32369652 PMC7541672

[157] Bradshaw, C.J.A., Leroy, B., Bellard, C., Roiz, D., Albert, C., Fournier, A. et al. (2016) Massive yet grossly underestimated global costs of invasive insects. Nat. Commun. 7, 12986 10.1038/ncomms12986 27698460 PMC5059451

[158] Wechsler, S. and Smith, D. (2018) Has resistance taken root in U.S. corn fields? demand for insect control. Am. J. Agric. Econ. 100, 1136–1150 10.1093/ajae/aay016

[159] Vorobieva, A.A., White, P., Liang, B., Horne, J.E., Bera, A.K., Chow, C.M. et al. (2021) De novo design of transmembrane β barrels. Science 371, eabc8182 10.1126/science.abc8182 33602829 PMC8064278

[160] Xu, C., Lu, P., Gamal El-Din, T.M., Pei, X.Y., Johnson, M.C., Uyeda, A. et al. (2020) Computational design of transmembrane pores. Nature 585, 129–134 10.1038/s41586-020-2646-5 32848250 PMC7483984

[161] Miehling, J., Goricanec, D. and Hagn, F. (2018) A split-Intein-Based method for the efficient production of circularized nanodiscs for structural studies of membrane proteins. ChemBioChem 19, 1927–1933 10.1002/cbic.201800345 29947468

[162] Nasr, M.L., Baptista, D., Strauss, M., Sun, Z.J., Grigoriu, S., Huser, S. et al. (2017) Covalently circularized nanodiscs for studying membrane proteins and viral entry. Nat. Methods 14, 49–52 10.1038/nmeth.4079 27869813 PMC5199620

[163] Bayly-Jones, C. (2021) Structural and functional characterisation of MACPF proteins in immunity

[164] Spicer, B.A. and Dunstone, M.A. (2021) Going full circle: determining the structures of complement component 9. Methods Enzymol. 649, 103–123 10.1016/bs.mie.2021.01.020 33712184

[165] Czajkowsky, D.M., Hotze, E.M., Shao, Z. and Tweten, R.K. (2004) Vertical collapse of a cytolysin prepore moves its transmembrane beta-hairpins to the membrane. EMBO J. 23, 3206–3215 10.1038/sj.emboj.7600350 15297878 PMC514522

